# Conditions affecting the association of general trait-anxiety with the ERN-Ne

**DOI:** 10.3389/fpsyg.2022.871443

**Published:** 2022-08-11

**Authors:** Vera Scheuble, Fee-Elisabeth Bertram, André Beauducel

**Affiliations:** ^1^Department of Methods and Diagnostics, Institute of Psychology, University of Bonn, Bonn, Germany; ^2^Department of Psychological Assessment and Differential Psychology, Insitute of Psychology, University of Kiel, Kiel, Germany

**Keywords:** ERN-Ne, general anxiety, worry, error aversiveness, gender moderation

## Abstract

The ERN-Ne of the event-related potential indicates error monitoring. Even though enlarged ERN-Ne amplitudes have often been related to higher anxiety scores, a recent meta-analysis provided very small effect sizes for the association of trait-anxiety with the ERN-Ne. Conditions modulating this association were investigated in the present study: (1) The generality of the trait-anxiety factor, (2) gender, and (3) experimental conditions, i.e., worry induction and error aversiveness. Participants (48% men) completed a flanker task. Worries were induced before the task by giving participants (*n* = 61) a bogus feedback claiming their responses were slower than the average responses of participants, whereas other participants (*n* = 61) got the feedback that they responded as fast as other participants. Aversiveness of errors was varied by playing sinus tones after too slow responses in one part of the task (no-scream condition) and aversive screams after too slow responses in another part (scream condition). Increased ERN-Ne amplitudes of response time errors occurred for individuals higher on trait-anxiety in the condition with induced worries and screams. A multiple group model for women and men indicated that women are more sensitive to conditions altering the association of trait-anxiety with the ERN-Ne.

## Introduction

The relationship between error monitoring and anxiety has been investigated to further understand state and trait anxiety from the perspective of cognitive psychology (Eysenck et al., 2007), clinical psychology (Cavanagh et al., 2017), and trait psychology (Moser et al., 2013). Error monitoring can be indicated by the error-related negativity (ERN-Ne), a negative deflection of the human event-related brain potential (ERP) that typically peaks about 80 to 100 ms after erroneous responses (Hohnsbein et al., 1991; Gehring et al., 1993). It has been proposed that the ERN-Ne amplitude indicates a neurobehavioral trait of defense reactivity, which may be related to psychopathology and sustained dispositional anxiety (Hajcak and Foti, 2008; Weinberg et al., 2012). Moser et al. (2013) performed a meta-analysis and estimated a correlation of *r* = −0.35 of anxious apprehension (worry) with the ERN-Ne. In a subsequent meta-analysis, Saunders and Inzlicht (2020) found that this correlation was partly due to publication bias and report a bias-corrected correlation estimate of *r* = −0.11 between the ERN-Ne and anxious apprehension. They encouraged future studies to collect data of large samples to be able to detect the association of the ERN-Ne with trait-anxiety. The effect size estimate provided by Saunders and Inzlicht (2020) implies that more than 500 participants are necessary to get a statistical power of .80 at a two-tailed alpha-level of *p* < 0.05 (G*Power 3.1; Faul et al., 2009). Such a large sample size is a challenge for a single ERP study and may encourage multi-center studies, cooperation of laboratories, and pooled-data approaches as, for example, the EEGManyLabs project (Pavlov et al., 2021) and the conscience project (Wacker, 2017). Although these projects are necessary in order to overcome problems of small effect sizes in ERP-research, single studies remain important because they help to maintain the statistical and methodological independence of results. Therefore, we further explore the conditions for the investigation of the association of trait-anxiety with the ERN-Ne amplitude within a single study.

### Generality of the association between the ERN-Ne and trait-anxiety

Based on the result that the association of the ERN-Ne with anxious apprehension was similar to the association of the ERN-Ne with mixed anxiety after bias-correction, Saunders and Inzlicht (2020) conclude that the relationship between anxiety and the ERN-Ne might be relatively general and non-specific. Anxiety has different facets including cognitive processes like worry, emotional, but also physiological processes like anxious arousal (Moser et al., 2013; Saunders and Inzlicht, 2020). This points to an investigation of the association of a general anxiety-trait with the ERN-Ne. However, Saunders and Inzlicht (2020) also emphasize that a broad profile of mixed anxiety comprising worry, anxious arousal, as well as depressive symptoms, and general discontent might blunt the relationship of anxiety with the ERN-Ne. General distress is a broad factor of negative affects reflecting whether a person tends to feel upset (Clark and Watson, 1991). It subsumes the tendency to have negative feelings like worry, fear, guilt, and sadness (Clark and Watson, 1991). It therefore incorporates negative affects, which are characteristic for both, anxiety and depression (Clark and Watson, 1991). This indicates that—even when a rather broad anxiety trait should be measured—the variance due to general negative emotionality or general distress might be controlled for. The State–Trait Anxiety Inventory (STAI; Laux et al., 1981) is a frequently used scale constructed to measure general anxiety. It aims to capture the cognitive and emotional components of anxiety (Laux et al., 1981). It should be noted that trait anxiety measured by the STAI has yet been related to a negative affect factor (Knowles and Olantunji, 2020), similar to general distress. Effects of an appropriate level of generality for optimal prediction are widely known (Wittmann and Klumb, 2006). A latent variable approach is suited to investigate a general anxiety-trait and to control for the effect of negative emotionality on the association of trait-anxiety with the ERN-Ne. Moreover, the specification of a general anxiety-trait helps to avoid the effects of multiple significance testing (Cribbie, 2007).

### The relationship between the ERN-Ne, anxiety, and gender

Furthermore, in the context of anxiety and error monitoring, possible effects of gender seem to be highly relevant. Women have more likely higher anxiety scores and tend to suffer more from it (Stavosky and Borkovec, 1987; Kroenke et al., 2007; McLean et al., 2011). Possible gender differences need to be unraveled to get a better understanding of anxiety and error monitoring, and they should be known for practical applications. In the meta-analysis by Moser et al. (2016), the relationship between symptoms of anxiety and the ERN-Ne was greater in women than in men (Moser et al., 2016). It is therefore expected that the association of the ERN-Ne with trait-anxiety is more pronounced for women. Even when the direction of this effect might depend on hormones, verbal mechanisms, the specificity or generality of trait-anxiety, and the role of verbally expressed worry, we follow Moser et al. (2016), who highlight that gender should be considered as a moderator on studies on the relationship between the ERN-Ne and anxiety.

### Experimental conditions that might enhance the association of the ERN-Ne with anxiety

Experimental conditions enhancing worry (Moser et al., 2013) or error aversiveness (Shackman et al., 2011; Proudfit et al., 2013) may also enhance the association of the ERN-Ne with trait-anxiety. Moser et al. (2013) proposed that worries distract from active goal maintenance during task performance followed by compensatory goal reactivation when errors occur, resulting in a more pronounced ERN-Ne. According to this theoretical approach, the induction of worries should reduce goal maintenance and should thereby enhance compensatory goal reactivation and ERN-Ne amplitudes during the task. Moser et al. (2013) classified several scales as measures of anxious apprehension (worry) or as measures of mixed anxiety, and expected that anxious apprehension (worry) is most closely associated to the ERN-Ne. Individuals scoring high on anxious apprehension (worry) could be more sensitive to the induction of worries by negative feedback of their task performance. If the relationship of trait-anxiety with the ERN-Ne is rather general and unspecific, a worry condition will also affect the correlation with general trait-anxiety. Another account explaining the association of the ERN-Ne with anxiety is based on the aversiveness of errors (Shackman et al., 2011; Proudfit et al., 2013). Experimental conditions increasing the aversiveness of errors may lead to a more pronounced ERN-Ne. The aversiveness of errors may be intensified by auditory aversive stimuli, such as loud screams following errors in a non-predictable way (Jackson et al., 2015; Mandrick et al., 2016; Patel et al., 2016). Thus, using unpredictable aversive auditory stimuli after errors may enhance the association of general trait-anxiety with the ERN-Ne. Although loud tones following errors may be regarded as a form of error feedback, they have been introduced as a punishment condition in previous ERN-Ne studies (Riesel et al., 2012; Riesel, 2019). According to aversive conditioning, the presentation of loud tones following errors produced a more pronounced ERN-Ne before feedback occurred (Riesel et al., 2012; Riesel, 2019). In accordance with conditioning studies (e.g., Riesel, 2019), the aversive tones are presented after wrong responses/errors and therefore also after the occurrence of the ERN-Ne. When participants are informed that too slow responses are followed by aversive tones and when they also experience the aversiveness of the tones after the first trials with too slow responses, they should form the conditioned expectation that the aversive tone follows slow responses. Due to the conditioned expectation of an aversive tone, errors should be more aversive and the ERN-Ne is expected to be more pronounced.

As high anxious individuals tend to hypervigilance and hyperactive error monitoring (Weinberg et al., 2010; Pasion et al., 2018), the ERN-Ne cannot be further enhanced by conditions enhancing error aversiveness in these individuals. Accordingly, the effect of error aversiveness should also be investigated under conditions allowing to reduce hypervigilance. Hypervigilance and hyperactive error monitoring of high anxious individuals may be reduced when they cannot completely exclude that their reaction was correct. Therefore, a condition resulting in error uncertainty will be introduced. Although Pailing and Segalowitz (2004) found that the ERN-Ne was attenuated by error uncertainty, the ERN-Ne has also been found for uncertain responses (Navarro-Cebrian et al., 2013). Navarro-Cebrian et al. (2013) proposed that the ERN-Ne may be related to response conflict rather than the specific detection of incorrect motor commands. This is compatible with the results of Kieffaber et al. (2016), suggesting that the ERN-Ne represents a general alarm elicited by the initiation rather than the commission of an error. They even propose that the ERN-Ne is unrelated to subjective error awareness. Stahl (2010) found that the ERN-Ne occurred for very late responses before error feedback. Accordingly, the ERN-Ne occurred when participants could not be completely sure of error commission. As uncertain responses are likely to induce response conflict, it might be possible to measure the ERN-Ne in the context of uncertain errors. Therefore, the relationship of the ERN-Ne, trait-anxiety, and error aversiveness was also investigated for uncertain errors in order to minimize ceiling effects resulting from hypervigilance.

### The present study

We investigated conditions that affect the association of trait-anxiety with the ERN-Ne. First, a more general trait-anxiety factor was investigated. It was expected that a more general trait-anxiety factor results in more pronounced correlations with the ERN-Ne than reported in Saunders and Inzlicht (2020). Second, the moderation of the association of trait-anxiety with the ERN-Ne by gender was investigated. It was expected that the correlation of trait-anxiety with the ERN-Ne is more pronounced for women than for men. Third, it was explored whether a worry condition and an error aversiveness condition enhance the correlation of the ERN-Ne with a general trait-anxiety dimension, especially in an error uncertainty condition that might reduce hypervigilance in high anxious individuals. It was, finally, investigated whether the relationship of trait-anxiety and ERN-Ne is altered when trait-anxiety is residualized for general distress variance.

## Materials and methods

### Participants

As the conditions investigated in the present study may help to overcome effect size limitations shown by Saunders and Inzlicht (2020), we performed power analysis on the basis of the effect size estimate provided by Moser et al. (2013). We used the R-script (pwrSEM v0.1.2) provided by Wang and Rhemtulla (2021) to estimate the power for the detection of a prediction of *r* = −0.35 by means of a latent independent variable based on four measured variables with loadings of 0.80 and a latent dependent variable based on three measured variables with loadings of 0.80. This implies that Cronbach’s Alpha of the latent independent variable is 0.88, whereas the internal consistency of the latent dependent variable is 0.84. For these values, the power to detect the effect of *r* = −0.035 at an alpha level of 0.05 (two-tailed) for *N* = 120 participants is 0.88. For less optimistic reliabilities based on loadings of 0.70 resulting in an independent variable internal consistency of 0.79 and a dependent variable internal consistency of 0.74, the resulting power is 0.79. A sample size of 120 participants was therefore regarded as a sufficient basis for further analysis.

Starting with data from 136 participants, data from 9 participants had to be excluded due to technical problems during EEG data collection. Five additional participants were excluded because they had no epochs for one of the four conditions (correct scream, correct no-scream, hand error scream, and hand error no-scream). Accordingly, a sample of 122 right-handed participants was available for data analysis (48% men; age: *M* = 23.97, *SD* = 3.90, range: 18–40 years; 53% examined at the University of Bonn and 47% at the University of Kiel), of which 61 participants were randomly assigned to the condition with no induced negative cognition and worries (NCW low) and 61 participants to the condition with induced negative cognition and worries (NCW high). The gender distribution did not differ across conditions (*p* = 0.47). Participants signed written informed consent before the examination and participated voluntarily. Participants received a reimbursement of 38€. The study was performed in accordance with the revised Helsinki Declaration (2013). The ethics board of the German Psychological Society approved the study.

### Measures

As described in the introduction, we aimed to measure a general anxiety factor. In order to tap the different facets of anxiety (including cognitive processes like worry, physiological processes like anxious arousal, feelings related to a general negative affect, and mixed anxiety), four anxiety scales were used. We selected scales that were applied in the context of ERN-Ne studies according to previous meta-analyses (Moser et al., 2013; Saunders and Inzlicht, 2020). The Behavioral Inhibition System (BIS) scale (Carver and White, 1994; Strobel et al., 2001) was used as a measure of anxious apprehension (worry). Moser et al. (2013) as well as Saunders and Inzlicht (2020) classified the BIS scale as an appropriate measure of anxious apprehension (worry). The anxious arousal scale from the Mood and Anxiety Symptom Questionnaire (MASQ; Watson and Clark, 1991) was used as a measure of anxious arousal. Furthermore, the General Distress scale of the MASQ was applied to measure negative affects, such as worry and feeling upset (Clark and Watson, 1991). We also used the Trait anxiety scale from the State–Trait Anxiety Inventory (STAI; Laux et al., 1981). The General Distress scale was used in two different ways, as a part of a broad mixed-anxiety factor and as a separate variable in order to residualize the mixed anxiety factor. Moreover, we applied the STAI-state, in order to perform a manipulation check for the worry condition. The three STAI-state items (7, 9, 17) that directly address worry (e.g., Item 17 “I am worried”) were aggregated so that the increase of the worry-state in the worry condition could be investigated.

### Experimental task

The current study was based on a flanker task that has successfully been used for the investigation of the ERN-Ne under conditions of high and low error certainty (Stahl, 2010). The task was presented *via* presentation V20.1 (Neurobehavioral Systems, Albany, NY) on a 19″ flat screen. The task consisted of numbers with three digits. Participants indicated whether the digit in the middle was even or odd. They were meant to press the right arrow key, when the number in the middle was even and the left arrow key, when it was odd. Participants were asked to respond as fast and correct as possible. The task started with an exercise consisting of 30 trials. The manipulation of the intensity of worries was based on a bogus feedback after the exercise. Participants were randomly assigned to one of two conditions: In the condition with induced worries (NCW high), participants got the feedback that response times were on average slower than the response times of all participants. In the condition without induced worries (NCW low) participants got the feedback that their response times were on average equal to the response times of all participants.

**Figure 1:**
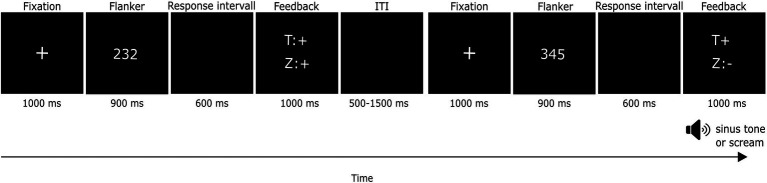
Trial sequence of the flanker task. In the first depicted trial, the correct button is pressed and the response is fast enough, whereas in the second depicted trial, the response is too slow and therefore a sound is played additional to the text feedback. The black screen was only presented when participants had not responded during the display of the number. T = button (German: Taste), Z = time (German: Zeit), + = correct, − = wrong.

The main task was split up into 10 blocks, each consisting of 50 trials. The blocks were separated by a 1-min break, which the participant could independently terminate (*cf.* Stahl, 2010). The manipulation of the aversiveness of errors was operationalized by a within-subject design. In the first 5 blocks, all participants heard a sinus tone when responding too slowly (low aversiveness of errors: no-scream condition). In the subsequent 5 blocks, either a scream (70% chance) or a sinus tone (30% chance) was played after too slow responses (high aversiveness of errors: scream condition). The median of the response times calculated over the 30 exercise trials subtracted with 10% of the median served as an individual response time criterion for the main task (*cf.* Stahl, 2010). The response time criteria of the participants ranged between 304.20 and 896.40 ms (*M* = 472.47 ms, *SD* = 81.17). The trial sequence is depicted in Figure 1. A trial started with a fixation cross (1,000 ms), followed by a three-digit number (900 ms). Responses were possible during the presentation of the number, or during a following black screen. When participants responded during the presentation of the number, the number display was presented for the whole 900 ms until its end, but the black screen did not follow it. When participants did not respond during the presentation of the number, the number display was followed by the black screen. The black screen always lasted 600 ms independent of the response time. Four different response types were possible: Participants could press the correct button fast enough (correct reaction). They could press the wrong button fast enough (hand error). They could press the correct button too slowly (RT error) and lastly, they could press the wrong button too slowly (double error). Hand errors can easily be detected by the participants so that hand errors are subjectively certain, whereas RT errors tend to be subjectively uncertain. After every trial, participants got feedback (lasting 1,000 ms) on whether they responded correctly and fast enough. The feedback consisted of the letters “T” and “Z” which stood for button (in German *Taste*) and time (in German *Zeit*), respectively, and the signs “+” and “-” standing for correct and wrong. The screams (84 dB) and sinus tones (84 dB, 500 Hz) for RT errors were played for 1,000 ms during feedback. In Stahl’s (2010) study, the mean of the slow responses was 610 ms, whereas the feedback/tones do not occur earlier than 900 ms after stimulus onset in the present task. It was therefore expected that the ERN-Ne typically occurs before the feedback and screams/tones of the aversive feedback condition. After the feedback, a black screen was presented (inter-trial-interval, ITI) for 500, 1,000, or 1,500 ms. The trials were presented in a pseudo-randomized order. A script (Presentation and Neurobehavioral Systems) of the experimental task can be obtained from the first author upon request.

### Procedure

Participants were recruited through announcements on bulletin boards, websites of the University of Bonn and the University of Kiel, and *via* mailing lists of students and former participants. In the announcements, the following selection criteria were given: Participants should be between 18 and 40 years old, right-handed, and be students. Exclusion criteria were neurological or mental illnesses, tinnitus, hypersensitivity to sounds, pregnancy, mood-altering medication, and the participation in a previous online study, in which the same questionnaires were tested. One day before the examination, participants were reminded to sleep as long as usual and to not consume alcohol or other stimulating substances. For the EEG examination, participants were seated in a sound-attenuated, electrical-shielded, and well-lit room. They completed the flanker task, which lasted about 45 min. Subsequently, participants were asked to complete the questionnaires described above. At the end of the experiment, participants were informed that the task feedback was faked, debriefed, and paid.

### EEG recording and quantification

**Figure 2:**
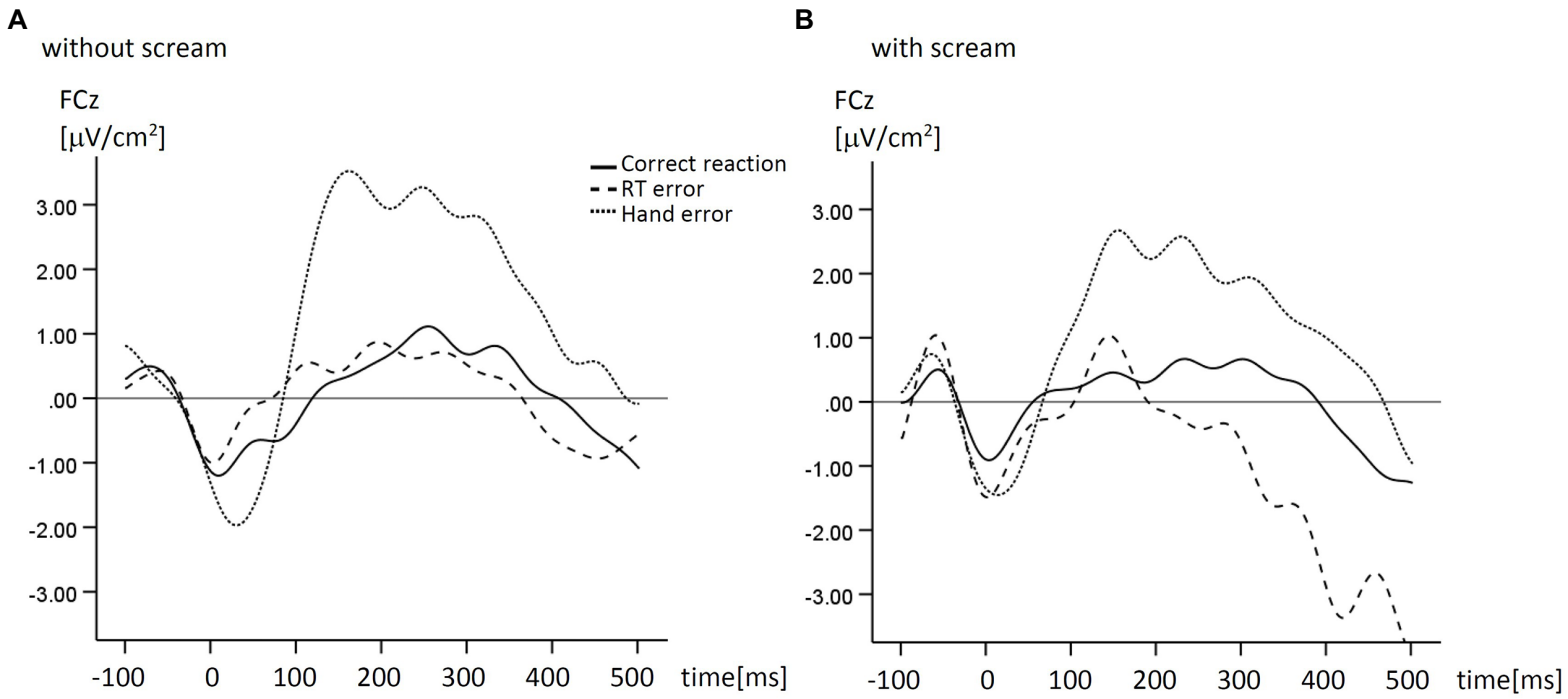
Mean response-locked ERP for the response types in the condition without scream **(A)** and in the condition with scream **(B)**. CSD transformations were performed.

**Figure 3:**
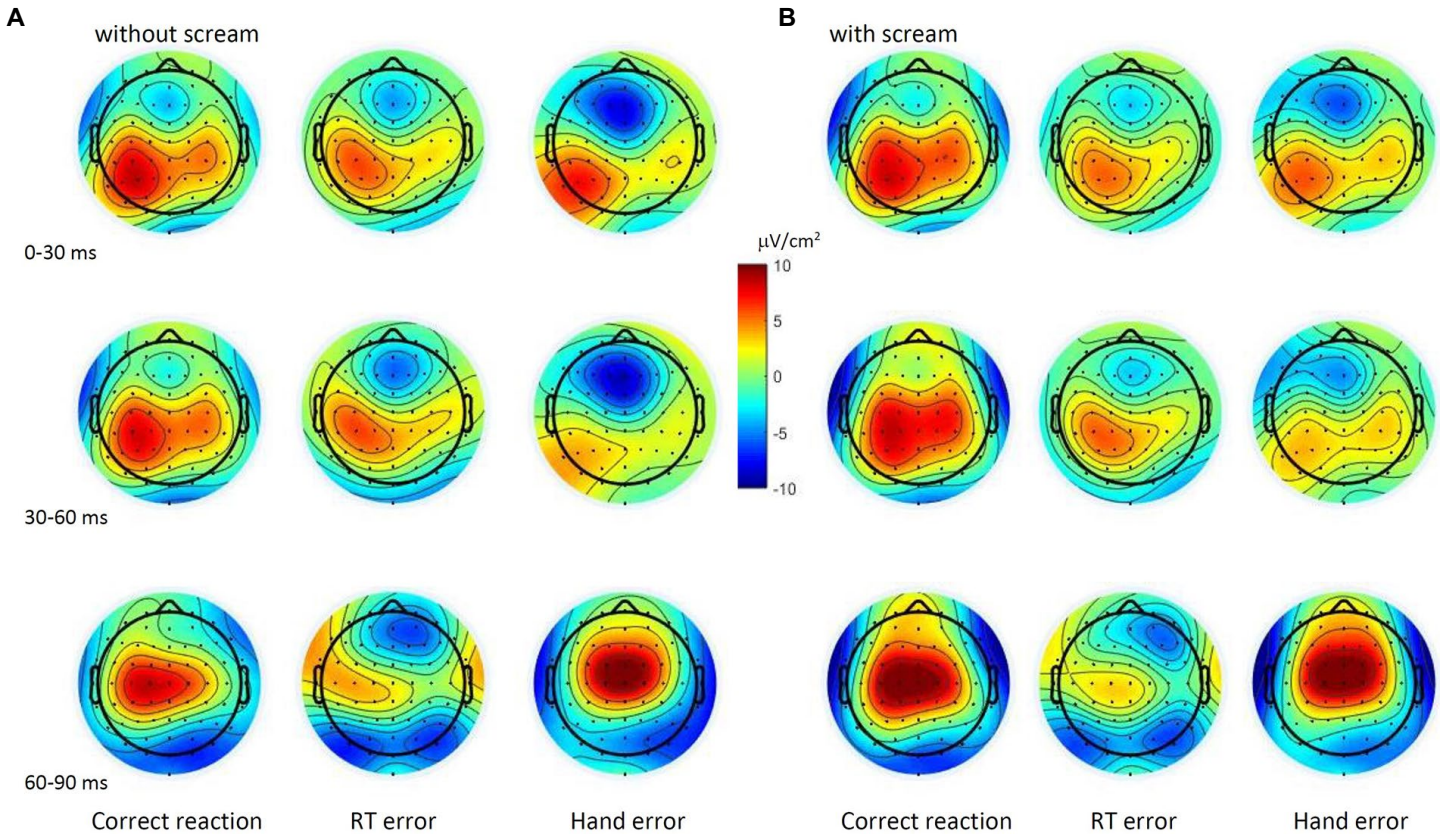
Topographic maps were determined for the response types as the mean activity within time windows of 30 ms in the condition without scream **(A)** and in the condition with scream **(B)**.

We used the ActiveTwo BioSemi system (BioSemi, Amsterdam, Netherlands) with 64 active scalp electrodes (10/10 system, Nuwer et al., 1998). The ground electrode was formed by the Common Mode Sense electrode and the Driven Right Leg electrode. The electrooculogram was recorded from two horizontal electrodes placed at the epicanthi of both eyes and one vertical electrode located approximately 1 cm below the right eye. Signals were digitized using Biosemi ActiView at a sampling rate of 512 Hz. Electrode offsets were kept within ±30 mV during EEG recording to ensure good contact between the electrodes and the scalp (Smith, 2007, p. 51). Offline analysis was performed with EEGLab (version 2019.1; Delorme and Makeig, 2004), based on MATLAB 7.14.739 (The MathWorks, Natick, MA). Data were re-referenced to the average signal of P9 and P10. We applied an off-line high-pass filter of 1 Hz and a low-pass filter of 15 Hz (Amodio et al., 2008). An independent component analysis (ICA; with infomax decomposition) based on 12 components was conducted to correct ocular artifacts. People with expertise in ocular artifact rejection selected components representing ocular artifacts based on a predefined scheme (i.a. frontal topography of the component and high amplitude variations) and these components were rejected from the dataset (c.f. Scheuble et al., 2019). Data were segmented into epochs spanning from 1,100 ms before to 500 ms after the response. We used an early baseline (−1,100 to −1,000 ms relative to the response) in order to provide a baseline correction independently from the current source density (CSD) analysis (performed for -100 ms to 500 ms). Furthermore, the baseline interval was set to a time before the mean reaction time (during the fixation cross) to ensure an ERP-neutral baseline that is not affected by the interested processes in order to provide clarity about the measured values (Brandeis and Lehmann, 2013). Epochs containing technical and muscle artifacts were rejected when the EEG signal of at least one of the electrodes exceeded ±85 μV. Following Stahl (2010), we performed current source density (CSD) transformation (Perrin et al., 1989) for ERP waveforms that occurred in a 600 ms response locked epoch (see Figure 2 for grand averages and Figure 3 for topography).

Because only 24 participants had at least 10 epochs for the double error category and the double errors were not relevant for our research hypotheses, this category was not considered for data analysis. However, even for some remaining categories (correct, hand error, RT error) the number of participants with less than 10 epochs was considerable. In order to avoid a substantial reduction of the sample resulting in reduced statistical power and to avoid variable sample sizes for each category, we performed SEM allowing for multiple imputation of missing values (Asparouhov and Muthén, 2010). Multiple imputation was based on 100 imputed datasets whenever the ERP of a participant was based on less than 10 epochs. We based the ERN-Ne analysis on data with at least 10 epochs because Olvet and Hajcak (2009) recommended that the ERN-Ne should be based on at least 6–8 epochs.

### Statistical analysis

We performed multiple imputation and SEM models with maximum likelihood estimation by means of Mplus 8.4 (Muthén and Muthén, 2019). In a first model, a general trait-anxiety factor based on trait BIS, Anxious Arousal, trait STAI, and General distress and the interaction term based on the corresponding anxiety scales with NCW were specified as independent variables. In a second model, the general anxiety factor was only based on trait BIS, Anxious Arousal, and trait STAI, and General distress was used in order to residualize the general trait-anxiety factor. In the second model, the interaction of general trait-anxiety with NCW was residualized for the interaction of General distress with NCW. In both models, four factors were specified as dependent variables based on the electrode positions Fz, FCz, and Cz, for the ERN-Ne amplitude for each of the conditions hand-error/no-scream, hand-error/scream, RT error/no-scream, and RT error/scream. The variances of the factors were fixed to one. Correlated errors were allowed between the measured ERN-Ne amplitudes to allow for common variances that are not represented by the factors. A dichotomous variable representing between-group effects was entered for NCW (high vs. low). The difference of the worry-related STAI-state items after the task minus the items before the task was also entered into the model. We specified a multiple-group model in order to investigate the path coefficients for women and men separately.

## Results

**Figure 4:**
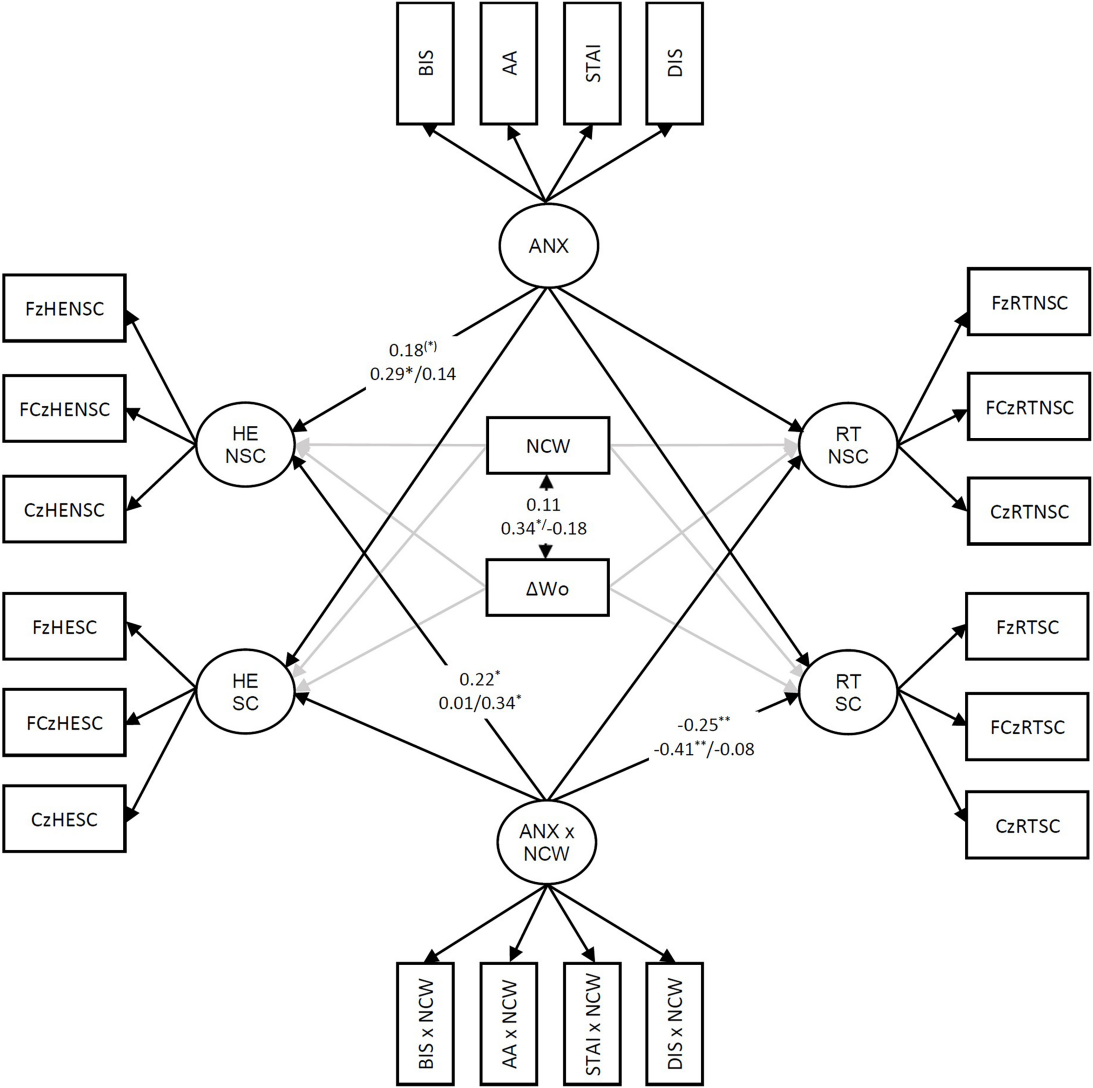
Model for the ERN-Ne and trait-anxiety including General distress (ANX); only coefficients that were significant in the total group or for men or women (completely standardized) path coefficients are given; before the slash: coefficients for women/behind the slash: coefficients for men; *p < 0.10, *p < 0.05, **p < 0.01, RT = RT error, HE = Hand error; SC = Part of the task with screams for RT errors; NSC = Part of the task without screams for RT errors; ΔWorry, Worry state before the task minus worry state after the task. BIS, trait BIS. AA, Anxious Arousal. DIS, General distress. STAI, trait-anxiety measured by the STAI. NCW, dummy variable representing the manipulation of worries; the inter-correlations of the predictors, factor loadings, and error terms are not presented. The gray arrows indicate that the path coefficients do not represent expected predictions and were only entered for the completeness of the model.

Results of behavioral data are given in the Supplement. The means of RT were between 352.22 ms and 664.81 ms (see Supplementary Table S1) so that the ERN-Ne mostly occurred before the feedback and aversive tones (not occurring before 900 ms after stimulus onset). The fit of the model for the investigation of the effects of a general trait-anxiety factor including General distress on the ERN-Ne (see Figure 4) was good (χ^2^_180_ = 221.94, *p* < 0.05, RMSEA = 0.044, CFI = 0.971, SRMR = 0.082). Significant path coefficients occurred for the factor representing the interaction of general trait-anxiety with NCW as a positive predictor of the ERN-Ne factor for hand-errors in the no-scream condition and as a negative predictor of the factor representing RT errors in the scream condition. For the general trait-anxiety factor, a marginal significant positive prediction of the ERN-Ne factor for hand errors in the no-scream condition was found. The fit of the multiple group model for women and men was acceptable (χ^2^_394_ = 468.03, *p* < 0.01, RMSEA = 0.055, CFI = 0.946, SRMR = 0.132). The relevant path coefficients are given in Figure 4. The correlation of NCW with the increase of indicated worries was significant for women, not for men (manipulation check based on worry items of the STAI). The positive prediction of the ERN-Ne on hand errors by general trait-anxiety was only significant for women. The positive prediction of hand errors by the interaction of general trait-anxiety with NCW was only significant for men. The negative prediction of the ERN-Ne for RT errors in the scream condition was only significant for women.

The fit of the model for the investigation of the effects of a trait-anxiety factor residualized for General distress on the ERN-Ne (see Supplementary Figure S1) was good (χ^2^_182_ = 221.26, *p* < 0.05, RMSEA = 0.042, CFI = 0.973, SRMR = 0.069). The main results were not substantially altered when compared with the model based on the factor including General distress in a general anxiety/negative emotionality factor. A notable difference between this model and the previous model was only that the interaction of trait-anxiety with NCW was a marginal significant positive predictor of the ERN-Ne on hand errors for men and a marginal significant positive predictor of the ERN-Ne for RT errors for men when the trait-anxiety was residualized for General distress.

## Discussion

The main result of the present study is that the interaction of general trait-anxiety with worry (NCW) was a negative predictor of the ERN-Ne on RT errors when they were followed by aversive screams. Accordingly, in the condition with induced worries and a higher aversiveness of errors, ERN-Ne amplitudes on RT errors were more pronounced for individuals with higher general trait-anxiety. This result was not altered when the interaction of trait-anxiety with NCW was residualized for the interaction of General distress (DIS) with NCW, indicating that this result may be primarily due to broad anxiety variance. Results were not altered when trait-anxiety, composed by BIS, AA, and STAI as measured variables was residualized for DIS, indicating that the component of negative affectivity that is also measured by these scales did not drive the present results. This is of special importance because Knowles and Olatunji (2020) found meta-analytic evidence that the STAI is primarily a measure of negative affectivity and not only a measure of trait-anxiety. In the present data set, the STAI has a higher correlation with DIS (*r* = 0.65) than with BIS (*r* = 0.60) and AA (*r* = 0.40), which is in line with the results of Knowles and Olatunji (2020). Therefore, the trait-anxiety factor of the present study also represents negative affectivity so that it is important that the results were not altered when DIS was partialled out.

The positive prediction of the ERN-Ne on hand errors by general trait-anxiety might be explained in a framework that considers that the ERN-Ne is a marker of expectancy violations (Holroyd and Coles, 2002). As negative feedback on too slow responses was also given in the no-scream condition, the participants were triggered towards fast responses, also because the threshold for negative feedback was related to their own performance. The focus on fast responding might have enhanced the expectation of hand errors. It is furthermore likely that higher trait-anxiety results in a stronger focus on fast responses, which might explain the positive prediction of the ERN-Ne by general trait-anxiety on hand errors. This interpretation is corroborated by the negative prediction of the ERN-Ne following RT errors in the scream condition by the interaction of trait-anxiety and NCW. When worries were induced and screams were given for RT errors, enlarged ERN-Ne amplitudes occurred on RT errors for individuals with higher general trait-anxiety. Because of the expectation of aversive screams following RT errors, high trait-anxiety participants in the NCW condition may have tried especially hard to avoid RT errors. In consequence, high trait-anxiety participants in the NCW condition may have experienced an especially strong prediction error resulting in a more negative ERN-Ne for RT errors. To sum up, the results of the ERN-Ne and trait-anxiety in the present study can be explained by a task that may have enhanced the expectation of hand errors and reduced the expectation of RT errors. Another explanation could be that the RT error condition reduced hypervigilance so that a more pronounced ERN-Ne with higher trait-anxiety in the NCW condition could only be found in this condition. The prediction of RT errors in the scream condition by the interaction of trait-anxiety with NCW can be related to RT error uncertainty. The RT error uncertainty together with the punishment condition provided by aversive screams after errors may have been especially aversive for high trait-anxious individuals in the NCW condition. The present results corroborate the results of previous studies showing an enhancement of the ERN-Ne in punishment conditions (Riesel et al., 2012; Meyer and Gawlowska, 2017; Riesel, 2019). Note that in these studies, as in the present study, the aversive stimuli were presented in a time interval after the ERN-Ne. Therefore, these studies do not show a direct effect of aversive stimuli on the ERN-Ne but an effect of the learned expectation that aversive stimuli follow an error on the ERN-Ne. In a broader sense, the present study provides further evidence of the sensitivity of the ERN-Ne to the motivational significance of errors (Hajcak et al., 2005). Moreover, the combined effect of negative performance feedback provided before the beginning of the task in the NCW condition, the punishment condition provided after errors in the scream condition, and RT error uncertainty may have been especially aversive for high trait-anxious individuals. However, whether RT error uncertainty itself is already an aversive condition or whether its aversive effects depend on the emotional and motivational context should be investigated in future studies.

Interestingly, a stronger positive prediction of the ERN-Ne by trait-anxiety was found for women than for men. This indicates that the effect of stronger associations of the ERN-Ne with anxiety that has been found for negative associations of anxiety with ERN-Ne (Moser et al., 2016) may also be found for positive associations. However, the induction of worry (NCW) resulted in a significant increase of worry for women, but not for men (manipulation check). This indicates that a more pronounced sensitivity of women to worry induction may not necessarily result in an enhanced ERN-Ne. Moreover, the more conventional result of a negative prediction of the ERN-Ne for RT errors in the scream condition by the interaction of trait-anxiety with NCW was also more pronounced for women. This is in line with the idea that the shift of expectations induced by the present task was more pronounced for women than for men. One may tentatively conclude that women are more sensitive to conditions inducing expectation shifts. Even when this conclusion should be investigated in further studies, the present results underline the recommendation of Moser et al. (2016) that research on the ERN-Ne should be based on samples comprising women and men.

### Limitations and future directions

A replication of the effects reported in the present study will be important to further our understanding of the conditions for positive and negative associations of trait-anxiety with the ERN-Ne. Moreover, it has been noted that the statistical power for the detection of a prediction by means of a number of separately measured predictors is typically larger than the power of a single prediction by means of a single latent predictor that is based on the same number of measured variables (Wang and Rhemtulla, 2021). Although this may be regarded as an argument in favor of a larger number of specific predictors, it should be considered that a large number of predictors may lead to alpha inflation, also in the context of SEM (Cribbie, 2007). Moreover, samples of about 500 participants or more as a basis for ERN-Ne research may be obtained in multi-center studies based on converging research designs, data processing, and statistical analysis (Wacker, 2017) in order to combine optimal statistical power with the advantages of latent trait modeling.

### Conclusion

In the present study, we found evidence for a negative prediction of the ERN-Ne on RT errors in the condition with screams by the interaction of general trait-anxiety with a worry condition (NCW). The negative prediction of the ERN-Ne on RT errors by the interaction of trait-anxiety with the worry condition was found when General distress variance was included in trait-anxiety as well as when trait-anxiety was residualized for General distress. This indicates that General distress did not blunt the relationship of ERN-Ne with general trait-anxiety. The positive prediction of the ERN-Ne on hand errors and the negative prediction of the ERN-Ne on RT errors were more pronounced for women than for men. This implies that the enhanced sensitivity of women to conditions that affect ERN-Ne-related processes does not depend on the direction of the association of the ERN-Ne with trait-anxiety. In a nutshell, the results of the present study imply that the conditions under which the association of trait-anxiety and ERN-Ne are investigated matter and have to be carefully considered in future studies, since differing associations between the ERN-Ne and anxiety can be found depending on them. Finally, our study results suggest that the conventional negative association between trait-anxiety and ERN-Ne amplitudes more likely occurs for (a) women, (b) when worries are prevalent, (c) errors are aversive, (d) hypervigilance can be excluded, and (e) when trait-anxiety is measured by a latent factor comprising several general anxiety scales.

## Data availability statement

The raw data supporting the conclusions of this article will be made available by the authors, without undue reservation.

## Ethics statement

The studies involving human participants were reviewed and approved by the ethics board of the German Psychological Society. The patients/participants provided their written informed consent to participate in this study.

## Author contributions

AB contributed to the funding acquisition. VS and F-EB collected data, supervised data collection, and prepared data. VS and AB performed statistical analyses and wrote the first draft of the manuscript. VS, AB, and F-EB wrote sections of the manuscript. All authors contributed to manuscript revision, read, and approved the submitted version.

## Funding

This study was funded by the German Research Foundation (DFG) to AL (LE 2240/6-1) and AB (BE 2443/11-1).

## Conflict of interest

The authors declare that the research was conducted in the absence of any commercial or financial relationships that could be construed as a potential conflict of interest.

## Publisher’s note

All claims expressed in this article are solely those of the authors and do not necessarily represent those of their affiliated organizations, or those of the publisher, the editors and the reviewers. Any product that may be evaluated in this article, or claim that may be made by its manufacturer, is not guaranteed or endorsed by the publisher.
