# Supplementary material for: Conditions affecting the association of general trait-anxiety with the ERN-Ne
Source: Front Psychol. 2022 Aug 11;13:871443. doi: 10.3389/fpsyg.2022.871443 (PMC9404500; doi:10.3389/fpsyg.2022.871443)
Supplement: Supplementary file 1 [file Data_Sheet_1.docx]

Supplementary Material

Conditions affecting the association of general trait-anxiety with the ERN-Ne

S1. Behavioral data

The statistical analyses for response times and response frequencies were performed with IBM SPSS (Version 25). Kolmogorov-Smirnov tests revealed that some distributions of response times and response frequencies were not normally distributed (*p* = .00‑.20). Therefore, we performed non-parametric tests for response times and response frequencies.

When multiple tests were required to follow up on significant results, the level of significance was adjusted by Bonferroni correction. The follow-up tests were each performed for three comparisons. Therefore, we used an alpha level of 0.05/3= 0.017 for these tests. Only two-tailed *p*-values are reported.

Descriptive statistics for response times and response frequencies are given in Table S1. The Friedman test for response frequencies revealed a significant effect of Response type (χ^2^_2_= 153.95; *p* < .001). Pairwise Wilcoxon tests yielded that correct responses were more frequent than RT errors (*T*= 6,986.50; *p* < .001) and hand errors (*T*= 7,495.00; *p* < .001). Furthermore, RT errors were more frequent than hand errors (*T*= 5,798.00; *p* < .001). Wilcoxon tests revealed that response frequencies differed between the scream and no-scream condition for correct responses (*T*= 7,426.00; *p* < .001), RT errors (*T*= 28.50; *p* < .001), and hand errors (*T*= 7,161.50; *p* < .001). Correct responses and hand errors were more frequent in the condition with screams compared to the condition without screams (Table S1). Conversely, RT errors were more frequent in the condition without screams than in the condition with screams (Table S1). Mann-Whitney tests revealed that the NCW effect was neither significant for correct responses, nor for RT errors, nor for hand errors (all *ps* > .35).

The Friedman test of response times yielded a significant effect of Response type (χ^2^_2_= 191.67; *p* < .001). Responses were slower for RT errors compared to hand errors (*T*= 7,503.00; *p* < .001) and correct trials (*T*= 7,503.00; *p* < .001). Responses were also slower for correct trials compared to hand errors (*T*= 1,813; *p* < .001). Corresponding to the results of the response frequencies, Wilcoxon tests revealed a significant difference between the response times of the scream and no-scream condition for RT errors (*T*= 2,452.00; *p* < .017), hand errors (*T*= 118.00; *p* < .001), and correct responses (*T*= 14.00; *p* < .001). Responses were slower in the no-scream, compared to the scream condition for correct trials and hand errors. Conversely, responses were slower in the scream compared to the no-scream condition for RT errors. The NCW effect was neither significant for RT errors, nor for hand errors, nor for correct responses (all *ps* > .53).

**Table S1.** Means and standard errors of means (S.E.M.) of response frequencies [%] and response times [ms] for the different response types in the condition of the task with and without screams.

|  | Condition  without screams, *M* (*S.E.M.*) | Condition  with screams, *M* (*S.E.M.*) |
| --- | --- | --- |
| Response frequency [%] |  |  |
| Correct response | 47.72 (1.35) | 62.34 (1.28) |
| RT error | 36.12 (1.40) | 15.58 (1.28) |
| Hand error | 11.31 (0.65) | 19.56 (1.00) |
| Double error | 4.84 (0.32) | 2.53 (0.24) |
| Response time [ms] |  |  |
| Correct response | 400.24 (6.48) | 359.11 (7.27) |
| RT error | 548.77 (8.46) | 554.06 (11.28) |
| Hand error | 394.34 (6.29) | 352.22 (7.01) |
| Double error | 591.49 (17.52) | 664.81 (24.99) |

*Note.* Since the response frequencies and response times did not significantly differ between the conditions with vs. without induced negative cognition/worries (NCW), the descriptive statistics are given for the whole sample.

NCW

∆Wo

BIS x NCW

AA x NCW

STAI x NCW

DIS x NCW

FzHENSC

FCzHENSC

CzHENSC

FzHESC

FCzHESC

CzHESC

FzRTNSC

FCzRTNSC

CzRTNSC

FzRTSC

FCzRTSC

CzRTSC

BIS

AA

STAI

DIS

0.14

0.29*/0.14

0.10

0.28^**/^-0.18

0.07

-0.14/0.25^(*)^

0.22^(*)^

0.01/0.34^*^

-0.26^**^

-0.41^**^/-0.08

0.14

-0.14/0.24^(*)^

**Figure S1.** Model for the ERN-Ne and trait-anxiety residualised for General distress variance, only coefficients that were significant in the total group or for men or women (completely standardized) path coefficients are given; before the slash: coefficients for women/behind the slash: coefficients for men; (*) p < .10, **p* < .05, ***p* < .01, RT = RT error, HE = Hand error; SC = Part of the task with screams for RT errors; NSC = Part of the task without screams for RT errors; ΔWorry: Worry state before the task minus worry state after the task; BIS = trait BIS; AA = Anxious Arousal; DIS = General distress; STAI = trait anxiety measured by the STAI; NCW = dummy variable representing the manipulation of worries; the inter-correlations of the predictors, factor loadings, and error terms are not presented. The gray arrows indicate that the path coefficients do not represent expected predictions and were only entered for the completeness of the model.
